# International food trade contributes to dietary risks and mortality at global, regional and national levels

**DOI:** 10.1038/s43016-023-00852-4

**Published:** 2023-10-09

**Authors:** M. Springmann, H. Kennard, C. Dalin, F. Freund

**Affiliations:** 1 Environmental Change Institute, Oxford University Centre for the Environment, University of Oxford, Oxford, UK; 2 Centre on Climate Change and Planetary Health, London School of Hygiene and Tropical Medicine, London, UK; 3 UCL Energy Institute, Bartlett School of Environment, Energy and Resources, University College London, London, UK; 4 Center on Global Energy Policy, Columbia University, New York, NY USA; 5 UCL Institute for Sustainable Resources, Bartlett School of Environment, Energy and Resources, University College London, London, UK; 6 Laboratoire de Géologie de l’ENS, UMR8538 du CNRS, PSL Research University, Paris, France; 7 Johann Heinrich von Thünen Institute, Federal Research Institute for Rural Areas, Forestry and Fisheries, Institute of Market Analysis, Braunschweig, Germany

**Keywords:** Risk factors, Sustainability, Geography

## Abstract

Food trade is generally perceived to increase the availability and diversity of foods available to consumers, but there is little empirical evidence on its implications for human health. Here we show that a substantial proportion of dietary risks and diet-related mortality worldwide is attributable to international food trade and that whether the contributions of food trade are positive or negative depends on the types of food traded. Using bilateral trade data for 2019 and food-specific risk–disease relationships, we estimate that imports of fruits, vegetables, legumes and nuts improved dietary risks in the importing countries and were associated with a reduction in mortality from non-communicable diseases of ~1.4 million deaths globally. By contrast, imports of red meat aggravated dietary risks in the importing countries and were associated with an increase of ~150,000 deaths. The magnitude of our findings suggests that considering impacts on dietary risks will become an important aspect of health-sensitive trade and agriculture policies, and of policy responses to disruptions in food chains.

## Abstract (web summary)

A large share of all food produced for human consumption is internationally traded, including foods with important public health implications such as fruits, vegetables, legumes, nuts and red meat. Using bilateral trade data and food-specific risk–disease relationships, this study estimates the impact of food imports on diet-related health effects per country.

## Main

About a quarter of all food produced for human consumption is internationally traded^1^. Trading food between countries is generally perceived to increase the supply, access and diversity of food available to consumers^2–4^ and, in principle, can contribute to greater food and nutrition security^5–11^, and a more efficient use of environmental resources^12^. However, concerns have been raised about the role food trade plays in outsourcing environmental pollution^13–16^ and the health risks associated with changing dietary patterns and increasing levels of overweight and obesity^17–20^.

Among the various approaches to studying the relationship between trade and health, there have been those focused on assessing the correlations between general markers of health (for example, life expectancy and body mass index) and either trade liberalization (often measured in terms of globalization and trade indices) or specific trade agreements (as captured by statistical or process-based economic models)^2,5,21^, as well as those focused on describing the contributions international trade has made to the distribution of calorie and nutrient availability in an attributional sense^8,10,11^. However, there has been little research on how the trade in food contributes to those dietary risks that are related to food intake and the associated diet-related diseases^22^.

Here we quantify the proportion of dietary risks and diet-related mortality that is attributable to international food trade. Dietary risks include eating too few fruits, vegetables, legumes and nuts and too much red meat (including beef, lamb, goat and pork)^23–25^. They are a leading cause of non-communicable diseases, such as heart disease, stroke, cancer and diabetes, and collectively responsible for one in five deaths globally^24,26,27^. Linking dietary risks to international food trade can help identify the role food imports play in the dietary health of the importing country and trace the responsibility for those impacts to the exporting country. We use this demand-driven attributional perspective to derive implications for health-sensitive food, trade and agriculture policies. Such policies have particular relevance in light of possible trade disruptions from domestic policies such as Brexit, natural disasters such as those from climate change and armed conflicts such as that between Russia and Ukraine.

For our analysis, we used detailed bilateral trade data^1^ and an algorithm that links food consumption with primary production^28^ to track the contribution food exports of one country made to national consumption in another country, and we used established risk–disease relationships^29–34^ together with mortality rates and population numbers^35^ to estimate the proportion of diet-related diseases and mortality that is attributable to traded foods (see Methods for further details). The contribution of food trade to a country’s diet is understood as the amount of dietary intake that stems from foods that are imported from other countries, representing the amount of the country’s food consumption that is imported rather than being sourced domestically. The imported portion of available foods was adjusted for food waste at the household level before the associated impacts on dietary risks and mortality were assessed^36^, and exports were always treated as exports, that is, excluded from national consumption.

Our analysis complements and differs from the economic analyses of trade scenarios that take into account economic feedbacks across regions and markets, and also from scenario analyses more generally. The aim of such analyses would be, for example, to analyse the relative impacts of trade versus no-trade scenarios or to quantify the changes in national and global markets to trade bans or liberalizations^37^. In our analysis, we are interested in quantifying what proportion of increases or decreases in dietary risks and diet-related diseases in each country can be attributed to imported foods, identifying the countries of origin of these foods and extending the attribution of positive or negative dietary-health impacts to those countries. This attributional perspective is similar to input–output assessments, for example, of the environmental impacts embodied in trade^13–16^, and complements those by assessing the dietary risks and associated health impacts embodied in food trade. Thus, our estimates do not constitute concrete scenarios of changes in trade but instead highlight the importance of considering the role food trade plays in dietary health irrespective of any particular policy measure.

## Results

**Extended Data Fig. 1:**
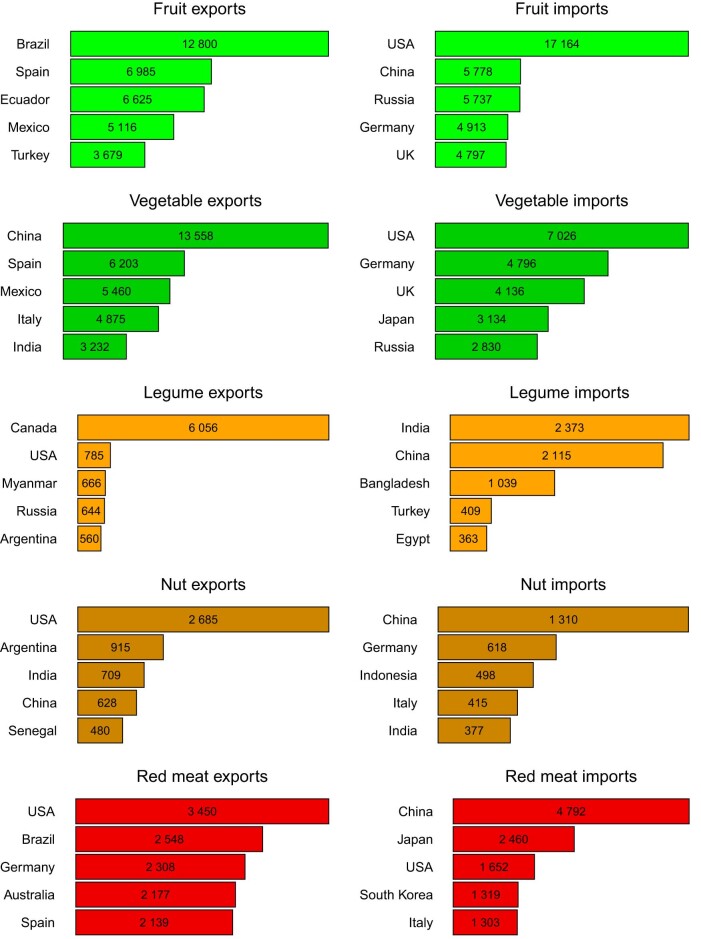
Leading exporters and importers of foods related to dietary risk, with trade flows measured in kilo tonnes per year (kt/yr). The foods include fruits, vegetables, legumes, nuts, and red meat.

According to our analysis, more than 190 million tonnes (Mt) of foods related to dietary risks, representing 3–12% of their production, was exported from one country to another in 2019 (Supplementary Table 6 and Extended Data Fig. 1). This included 86 Mt (11% of production) of fruits, 58 Mt (5%) of vegetables, 25 Mt (11%) of red meat, 12 Mt (3%) of legumes and 8 Mt (12%) of nuts. Most fruits, legumes and nuts were exported from the Americas (42 Mt (27%), 8 Mt (3%) and 4 Mt (48%), respectively), especially Brazil and Argentina; most vegetables from Asia (22 Mt, 2%), especially China; and most red meat from Europe (12 Mt, 25%), especially Germany.

Food imports contributed an average of 3–31 grams per person per day (g d^−1^) to national food availability, representing 5–21% of demand (Supplementary Table 7 and Extended Data Fig. 1). The amount of per-person food demand met by imports was 31 g d^−1^ for fruits (14% of demand), 21 g d^−1^ for vegetables (5%), 9 g d^−1^ for red meat (11%), 4 g d^−1^ for legumes (19%) and 3 g d^−1^ for nuts (21%). By region, the amount of food demand met by imports ranged from 4 g d^−1^ (2%) of fruits in Africa to 145 g d^−1^ (64%) in Europe, 7 g d^−1^ (4%) of vegetables in Africa to 94 g d^−1^ (32%) in Europe, 2 g d^−1^ (29%) of legumes in Oceania to 8 g d^−1^ (100%) in Europe, 1 g d^−1^ (5%) of nuts in Africa to 12 g d^−1^ (97%) in Europe and 1 g d^−1^ (4%) of red meat in Africa to 34 g d^−1^ (23%) in Europe.

**Fig. 1:**
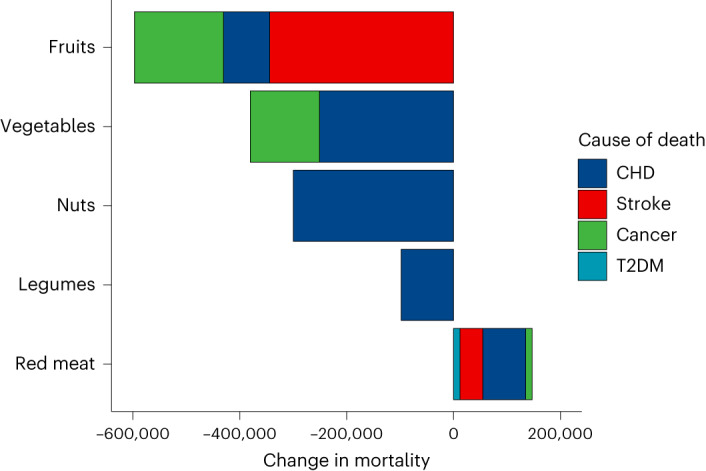
Contribution of traded foods to the diet-related disease burden in the importing countries by dietary risk and disease. Dietary risks include low intake of fruits, vegetables, nuts and legumes, and high intake of red meat. Diseases include coronary heart disease (CHD), stroke, cancer and type 2 diabetes mellitus (T2DM).

The trade-related contributions in food intake (after subtracting food waste) were associated with a net reduction in diet-related mortality of 1.2 million deaths (95% confidence interval, 0.8–1.7 million; Fig. 1). About half of the avoided deaths (53%) were from coronary heart disease, and a quarter each from stroke (25%) and cancer (23%). The trade-related contributions to fruit intake were responsible for the largest reductions in mortality (−597,000), followed by vegetables (−380,000), nuts (−300,000) and legumes (−98,000). By contrast, the trade-related contributions to red meat intake were associated with an increase in diet-related mortality (+147,000).

**Fig. 2:**
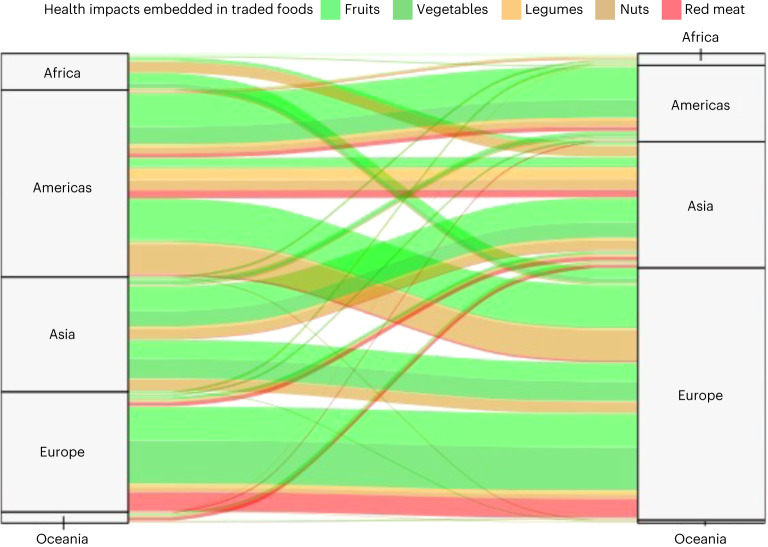
Trade flows of dietary risks, measured in changes in mortality, between exporting and importing regions. Changes in mortality occur and are estimated in the importing region (right) and traced back to the exporting region (left) to highlight the connection via trade. Please note that the trade flows of diet-related mortality impacts are not strictly conserved between exporting and importing regions. Mortality impacts would differ if the exported foods would be consumed in the exporting country owing to differences in baseline intake and mortality rates.

Of the total reductions in diet-related mortality, more than half were associated with food imports to Europe (−675,000; 55%), especially fruits exported from the Americas and vegetables from other parts of Europe (Fig. 2 and Supplementary Table 8). This was followed by imports to Asia (−301,000; 25%) and the Americas (−209,000; 17%), each case driven by fruits and vegetables exported from within the region. Smaller proportions were associated with imports to Africa (−33,000; 3%) and Oceania (−7,000; 1%), including vegetables from Asia and Europe. When attributing health impacts to the exporting region, the Americas were the largest contributor to diet-related reductions in mortality (−507,000; 41%), followed by Asia (−365,000; 30%), Europe (−231,000; 19%), Africa (−118,000; 10%) and Oceania (−5,000; 0.4%).

**Fig. 3:**
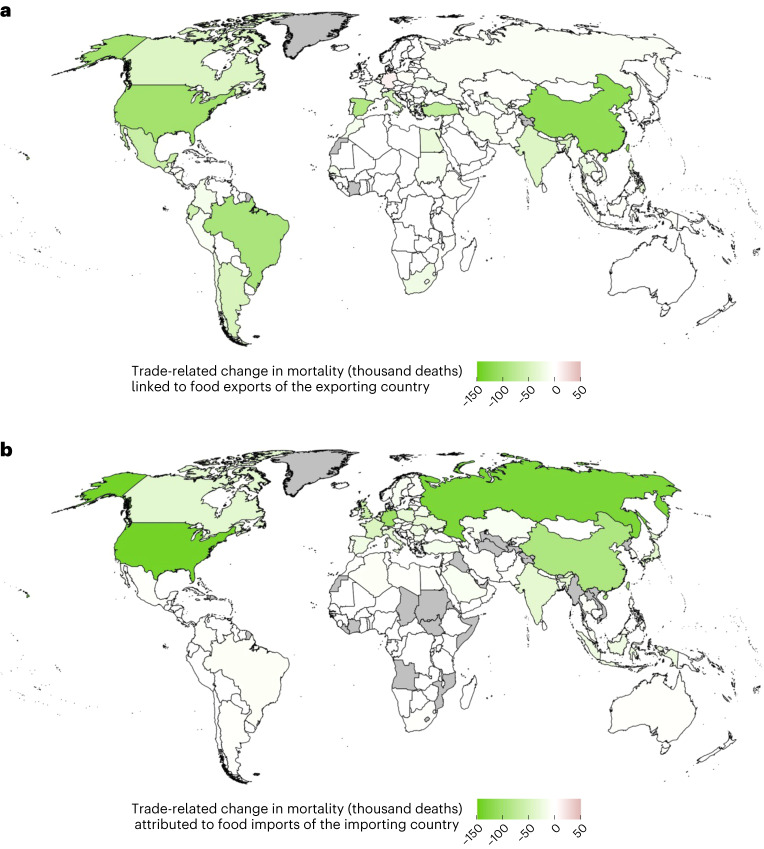
Exporters and importers of dietary risks, measured in changes in mortality. **a**,**b**, Changes in mortality occur and are estimated in the importing region (**b**) and traced back to the exporting region (**a**) to highlight the connection via trade.

**Fig. 4:**
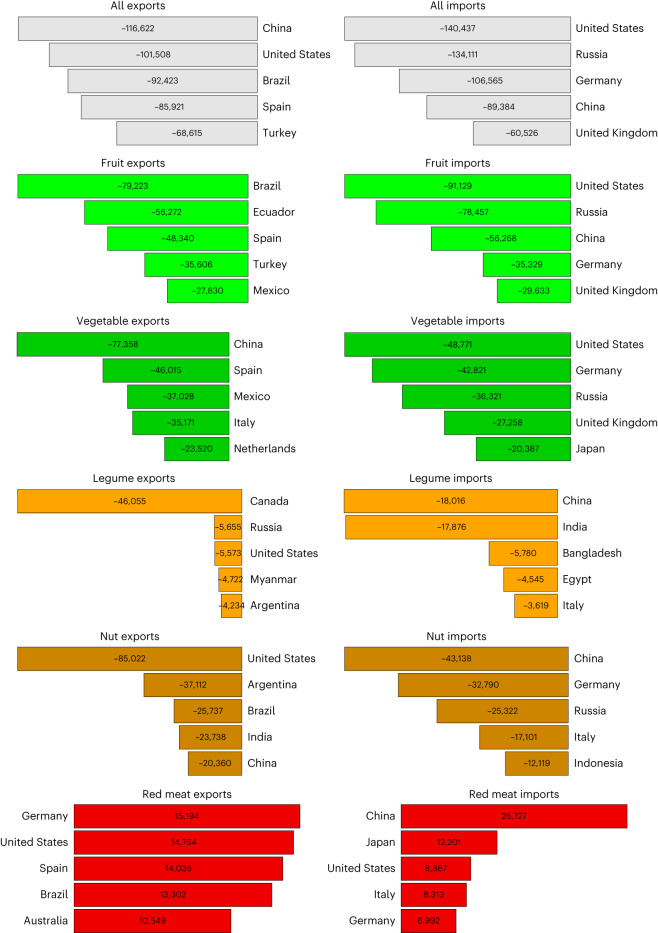
Leading importers and exporters of dietary risks by food group, measured in changes in mortality. Changes in mortality occur and are estimated in the importing region and traced back to the exporting region to highlight the connection via trade.

**Extended Data Fig. 2:**
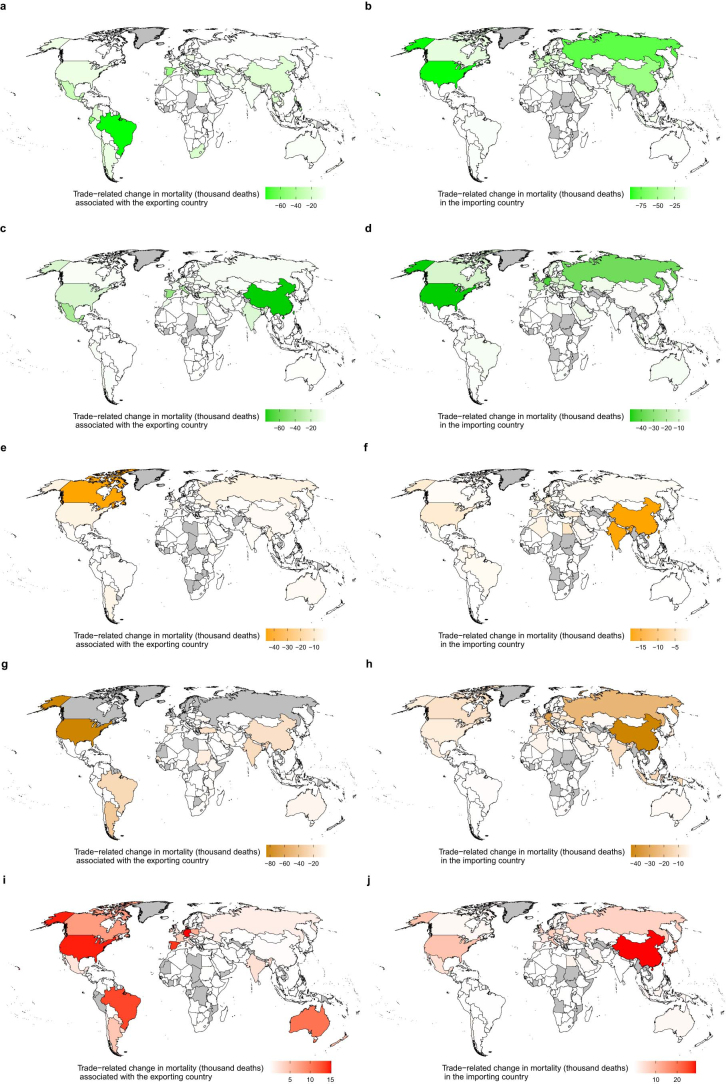
Exporters and importers of dietary risks related to foods, measured in changes in mortality. The foods include fruits (**a, b**), vegetables (**c, d**), legumes (**e, f**), nuts (**g, h**), and red meat (**i, j**).

At the country level, imports of health-sensitive foods (that is, foods related to dietary risks) contributed to health benefits in 152 out of 153 importing countries (Fig. 3 and Extended Data Fig. 2). The countries with the greatest health benefits, driven to large degrees by imports of fruits and vegetables, were the United States (−140,000), Russia (−134,000), Germany (−107,000), China (−89,000) and the United Kingdom (−61,000). The same set of countries also benefited from imports of nuts and legumes; other leading beneficiaries included Italy and India for both nuts and legumes, and Bangladesh and Egypt for legumes (Fig. 4). The only country exhibiting a net increase in diet-related mortality from trade was Papua New Guinea (+4 deaths) where the negative health impacts associated with imports of red meat exceeded the positive impacts of other food imports.

Out of 181 countries that exported health-sensitive foods, 162 (90%) contributed to reductions in diet-related mortality through their exports and 19 (10%) to increases (Fig. 3 and Extended Data Fig. 2). The countries whose exports contributed most to a reduction in diet-related mortality were China (−117,000), driven by vegetables and nuts; the United States (−102,000), driven by nuts and legumes; Brazil (−92,000) and Spain (−86,000), both driven by vegetables and fruits; and Turkey (−69,000), driven by fruits. Other leading exporters were Ecuador and Mexico for fruits, Italy and the Netherlands for vegetables, Argentina and India for nuts and Canada for legumes (Fig. 4). The countries that contributed to net increases in mortality through high exports of red meat included Germany (+10,000), Denmark (+7,000), Ireland (+3,500), Uruguay (+2,000) and Paraguay (+1,400).

## Discussion

We quantified the contribution of international food trade to five dietary risks and associated mortality. We found that international trade in fruits, vegetables, legumes and nuts contributed to improved dietary risks in the importing countries, which was associated with a reduction in mortality from non-communicable diseases of 1.4 million deaths globally. By contrast, trade in red meat contributed to aggravated dietary risks in the importing countries and was associated with an increase of 147,000 deaths. The net change in mortality attributable to food trade amounts to a fifth (19%) of the total diet-related health burden that is associated with eating too few fruits, vegetables, legumes and nuts, and too much red meat^38^. Our analysis implies that the trade in foods makes substantial contributions to the magnitude and distribution of dietary risks worldwide.

Our study has several strengths that advance the current literature on health and trade. First, it links food trade to final health outcomes instead of considering markers of dietary health^22^. Second, it explicitly resolves trade patterns instead of considering indices of trade openness^21^. Third, it provides country-level analyses for all countries participating in international trade instead of focusing on specific regions. Fourth, our method of linking food trade to dietary risks and associated mortality is less time and context dependent than existing regression analyses^39^, and can be flexibly applied in future research, including in longitudinal studies of trade and analyses of past and future trade agreements^37^.

Our study is also subject to several caveats. First, our analysis covered major dietary risks, but it did not analyse the impacts food trade can have on other aspects important for health. These include the impact food trade has for overweight and obesity in the importing countries^17–19^ or the relationship between food trade and consumption of ultra-processed foods high in sugar, salt and fats^40,41^. Process-based analyses of these and further health aspects related to trade are an important avenue for future research^21,22^. As such, our study cannot determine whether food trade is generally beneficial or detrimental to health. In particular, the trade in ultra-processed foods can be expected to be associated with increases in weight-related risks in the importing country^40,41^ and therefore counteract the positive contributions of importing health-promoting foods that reduce dietary risks.

Second, our study is subject to caveats that apply to comparative risk assessments and nutritional epidemiology^42^. In particular, our health analysis is based on the assumption that the risk–disease relationships we used to link changes in dietary risks to mortality describe causal associations. This assumption is supported by the existence of statistically significant dose–response relationships in meta-analyses, the existence of plausible biological pathways and supporting evidence from experiments, for example, on intermediate risk factors^29–34^. However, residual confounding with unaccounted risk factors cannot be ruled out entirely in epidemiological studies.

Our study adds to the body of evidence suggesting that food trade can play both positive and negative roles in health. Past analyses have quantified not only trade’s positive impact on nutritional adequacy, especially in high- and middle-income countries^8^, but also its role in increasing obesity, especially in low- and middle-income countries^20^. Our findings suggest that when it comes to foods related to dietary risks, trade plays a largely positive role, especially for regions with substantial imports such as Europe, the Americas and Asia. However, exceptions also exist, especially when focusing on the negative health impacts associated with exports of red meat, most of which originated from European and Latin American countries.

At a conceptual level, our analysis is related and adds a new health aspect to the literature on the environmental and social impacts embodied in trade flows^13–16^. In contrast to the existing literature, our study has found that the embodied health impacts affect the importing country while the environmental and social impacts affect the exporting one. However, similar to the existing literature, our study has also found that it is predominantly developed countries that benefit from importing, in our case, health-promoting foods from less developed countries (Supplementary Table 9). Another shared characteristic is that the embodied impacts are not preserved between exporting and importing regions. In our case, consuming the exported foods in the exporting regions would have different impacts as consuming them in the importing region owing to differing mortality rates and baseline consumption, even though all regions are currently consuming too few health-promoting foods^43^.

Our findings have several implications relevant to food, trade and agricultural policy. The data on trade in dietary risks can help plan trade agreements and understand trade exposure. For example, we found that Europe was the largest net beneficiary of trade in dietary risks, whereas the Americas were the largest net contributor, and also, Africa exported more dietary risks than it imported—in its case three times more (Supplementary Table 6). Our analysis suggests that disruptions in the trade of foods associated with dietary risks can substantially affect the burden of diet-related diseases, especially in countries that are heavily import dependent. Such disruptions can be the result of natural disasters related, for example, to climate change^44^, nationalistic policies that increase trade barriers such as the United Kingdom’s exit from the European Union^37^ or armed conflicts, provided they are sustained for prolonged periods^45^.

**Fig. 5:**
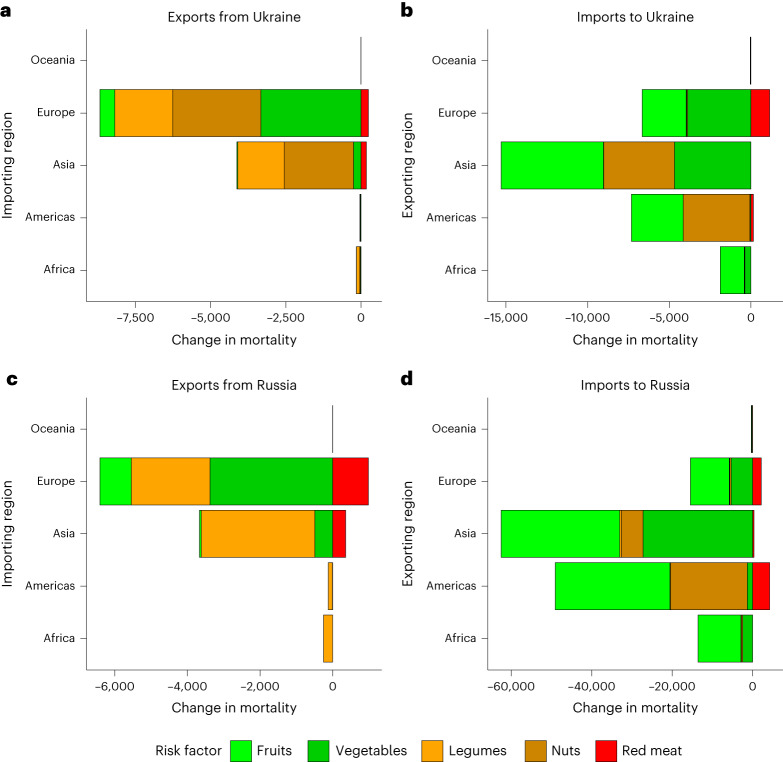
Trade-related changes in mortality, measured in number of deaths, by risk factor and region linked to Ukraine’s and Russia’s food exports and imports. **a**, Exports from Ukraine. **b**, Imports to Ukraine. **c**, Exports from Russia. **d**, Imports to Russia.

A particularly recent example of disruptions in food trade is the Russian invasion of Ukraine in 2022. Both Ukraine and Russia are major exporters of grains, and a shortfall in their exports could impact global wheat prices and food security^46^. Our analysis indicates that the health implications of changes in their trade of foods related to dietary risks can be important too (Fig. 5). We found that Ukrainian exports contribute to a net reduction in mortality in importing countries of 12,600 deaths (most of which are associated with nuts, legumes and vegetables), which is at risk owing to the Russian invasion. Russia, however, is one of the main beneficiaries of importing health-promoting foods—associated with 134,000 less deaths (most of which are associated with fruits, vegetables and nuts)—and therefore risks harming the health of their population should international sanctions include agricultural exports. Mitigating the impacts on trade in such foods could alleviate some of the indirect health consequences that this conflict could otherwise have.

Appropriate trade and agricultural policies can contribute to safeguarding the health benefits of trade, while minimizing its harm. Reducing tariffs on the export and import of health-promoting foods such as fruits, vegetables, legumes and nuts could ensure that populations have access to a variety of foods critical to good health^37^. At the same time, the detrimental health impacts from the export of foods linked to increases in mortality could be reduced by increased tariffs and appropriate agricultural policies in the exporting country. In countries we identified as net exporters of foods linked to increased mortality, such as Germany and Denmark, agricultural policies would be warranted that incentivize a transition towards greater diversification of production, instead of the current specialization on livestock production for export. As food trade is an important contributor to changes in dietary risks and mortality, safeguarding the trade in health-promoting foods and limiting those of unhealthy ones will be important aspects of trade and agricultural policies.

## Methods

To track food trade between countries, we made use of detailed bilateral trade data provided by the Food and Agriculture Organization of the United Nations (FAO)^1^. The FAO collects and processes the data according to the standard International Merchandise Trade Statistics methodology. It is based on source data provided by the United Nations Statistics Division, Eurostat and other national authorities. The FAO has checked the source data for outliers, added data on food aid and built statistical models to derive estimates for non-reporting countries and to fill data gaps. The trade database includes all food and agricultural products imported and exported annually by country.

In the bilateral trade data provided by the FAO, the source country is usually the country where the last value-added production step has taken place. For example, when a country imports raw material, processes it and re-exports the product, it will be listed as the source country. We used a balancing algorithm based on input–output accounting to clearly link the final demand to the origin of the primary product^28,47^. The algorithm is based on production data of primary products, bilateral trade data of primary products and the secondary products derived from them (for example, oils), and conversion factors for converting secondary products into primary equivalents based on caloric content and using extraction rates (Supplementary Tables 1 and 2).

We aggregated the commodity-level detail to food groups relevant to health analyses, including fruits, vegetables, legumes, nuts and red meat (Supplementary Table 3). We focused on those food groups for which disease associations have been identified in meta-analyses of epidemiological cohort studies^29–34^, but note that other types of traded food (for example, ultra-processed foods) can also have implications for health (for example, through their effect on weight levels). To analyse the health implications of traded foods, we converted the traded quantities into an equivalent change in per capita consumption by dividing by population numbers and subtracting the proportion of food waste that occurs at the household level^36^.

We developed a comparative risk assessment of dietary risks and used it to quantify the health implications of trade in food commodities. The comparative risk assessment included five dietary risks (fruits, vegetables, legumes, nuts and red meat) and their relationship to five disease end-points (coronary heart disease, stroke, colorectal cancer and type 2 diabetes). The relative-risk estimates that relate the risk factors to the disease end-points were adopted from meta-analyses of prospective cohort studies (Supplementary Table 4)^29–34^, and mortality and population data by age group and country were adopted from the Global Burden of Disease project^35^.

The selection of risk–disease associations used in the health analysis was supported by available criteria used to judge the certainty of evidence, such as the Bradford Hill criteria used by the Nutrition and Chronic Diseases Expert Group^23^ and the World Cancer Research Fund criteria used by the Global Burden of Disease project^24^, as well as NutriGrade (Supplementary Table 5)^25^. The certainty of evidence supporting the associations of dietary risks and disease outcomes used here was graded as moderate or high with NutriGrade^32–34^ and/or assessed as probable or convincing by the Nutrition and Chronic Diseases Expert Group^23^ and the World Cancer Research Fund^48^.

As our analysis was primarily focused on mortality from chronic diseases, we focused on adults aged 20 years or older, and we adjusted the relative-risk estimates for attenuation with age based on a pooled analysis of cohort studies focused on metabolic risk factors^49^, in line with other assessments^23,50^. In the uncertainty analysis, we used the low and high values of the 95% confidence intervals of the relative-risk estimates and standard methods of error propagation to derive confidence intervals of our estimates of trade-related changes in mortality. Our reporting follows the Guidelines for Accurate and Transparent Health Estimates Reporting (GATHER; Supplementary Information reporting file)^51^.

### Reporting summary

Further information on research design is available in the Nature Portfolio Reporting Summary linked to this article.

### Supplementary information

Supplementary Information Bilateral trade data, comparative risk assessment, Supplementary Results, Tables 1–9, References and GATHER checklist.

Reporting Summary

Supplementary Data 1 Supplementary data file containing the global, regional and national estimates of food trade, its impacts on food availability and intake and the associated health impacts.

## Data Availability

All data produced in this study are available as a supplementary data file available on figshare via https://figshare.com/s/24b15c6b93caad07a758 and with the digital object identifier 10.25446/oxford.24085362.

## Code availability

The codes for the trade and health analyses are described in detail in Supplementary Information and the references cited therein. They are available upon request.
