# Supplementary material for: International food trade contributes to dietary risks and mortality at global, regional and national levels
Source: Nat Food. 2023 Oct 9;4(10):886–93. doi: 10.1038/s43016-023-00852-4 (PMC10589094; doi:10.1038/s43016-023-00852-4)
Supplement: Supplementary file 1 — Bilateral trade data, comparative risk assessment, Supplementary Results, Tables 1–9, References and GATHER checklist. [file 43016_2023_852_MOESM1_ESM.pdf]

nature food

Article https://doi.org/10.1038/s43016-023-00852-4

International food trade contributes to dietary risks and mortality at global, regional and national levels

In the format provided by the authors and unedited

Table of Contents

Bilateral trade data 2

Comparative risk assessment 16

Supplementary results 19

Supplementary References 23

GATHER checklist...25

Bilateral trade data

For tracking food trade between countries, we made use of detailed trade data provided by the FAO. The dataset is collected, processed and disseminated by FAO according to the standard International Merchandise Trade Statistics (IMTS) Methodology. It is based on source data provided by UNSD, Eurostat, and other national authorities as needed. This source data is checked by FAO for outliers, trade partner data is used for non-reporting countries or missing cells, and data on food aid is added to take into account total cross-border trade flows. The trade database includes all food and agricultural products imported and exported annually by all the countries in the world.

In order to calculate the movement of food groups and agricultural products worldwide, we converted secondary processed foods into primary crop and animal product equivalents using values collated by Dalin and colleagues that were based on FAO product categorisation (SI Table 1).[1] Next, we used an algorithm developed by Kastner and colleagues which corrects FAO trade values to account for re-export of goods, and applied it to the FAO production and trade values.[2] This algorithm removes re-exports and allows the originating country of the product in question to be attributed the correct production quantity. This process provides a second-order correction to trade flows, with largest impacts in global hub countries such as the Netherlands, which import and re-export large quantities of agricultural products.

The following provides a summary of the trade-correction algorithm. Its structure is analogous to approaches taken in input-output analyses, where matrices of economic relations are converted into a form which shows the underlying reliance between sectors or regions. The derivation here is reproduced from Kastner and colleagues with additional comments for clarification.[2]

The goal of the analysis is to calculate the corrected trade matrix $\bar{\mathbf{R}}$, which is composed of the proportion of the domestic material input which is produced in another country. It is assumed that goods are consumed in a country in the same proportion as the imports and domestic production. The domestic material input (DMI) can be calculated from production and trade data by the following equation:

$$\mathbf{x} = \mathbf{p} + \mathbf{Z} \cdot \mathbf{i}$$

where $\mathbf{x}$ is the vector of DMI, $\mathbf{p}$ the production vector, and $\mathbf{Z}$ the bilateral trade matrix, which is multiplied by a vector of ones. Each row of each vector or matrix in the following corresponds to a specific country. The calculation is repeated for each year and commodity considered, with the commodities converted to primary equivalents (using the values in SI Table 1).

The first order case begins by assuming that the consumption (production plus imports) of a country in a given year, of a given commodity, is given by:

$$\mathbf{R^A} = \hat{\mathbf{p}} + \mathbf{Z}$$

where $\hat{\mathbf{p}}$ is a matrix containing the elements of the production vector $\mathbf{p}$ on the diagonal.

Following the central assumption of this approach, the matrix of shares of exports in DMI according to the country of destination is:

$$\mathbf{A} = \mathbf{Z} \cdot \hat{\mathbf{x}}^{-1}$$

Substituting this into the previous equation, and expanding the terms gives:

$$\mathbf{R}^{\mathbf{A}} = \hat{\mathbf{p}} + \mathbf{A} \cdot \hat{\mathbf{p}} + \mathbf{A} \cdot (\hat{\mathbf{x}} - \hat{\mathbf{p}}).$$

Here, the second and third terms presents the bilateral trade matrix split into two parts, corresponding to the exporting countries' production and the goods imported by the exporting country for other countries. The second order approximation of the corrected DMI then follows as:

$$\mathbf{R}^{\mathbf{B}} = \hat{\mathbf{p}} + \mathbf{A} \cdot \hat{\mathbf{p}} + \mathbf{A} \cdot \mathbf{A} \cdot \hat{\mathbf{p}} + \mathbf{A} \cdot \mathbf{Z}$$

and splitting the matrix $\mathbf{Z}$ again yields:

$$\mathbf{R}^{\mathbf{B}} = \hat{\mathbf{p}} + \mathbf{A} \cdot \hat{\mathbf{p}} + \mathbf{A} \cdot \mathbf{A} \cdot \hat{\mathbf{p}} + \mathbf{A} \cdot \mathbf{A} \cdot (\hat{\mathbf{x}} - \hat{\mathbf{p}})$$

This process can be continued to create further refinements towards the corrected matrix $\mathbf{R}$, which converges on the following form:

$$\mathbf{R} = \lim_{s \to \infty} \left(\sum_{n=0}^{s} \mathbf{A}^{n} \right) \cdot \hat{\mathbf{p}} + \lim_{s \to \infty} \mathbf{A}^{s} \cdot \mathbf{Z} = (\mathbf{I} - \mathbf{A})^{-1} \cdot \hat{\mathbf{p}}$$

The matrix $(\mathbf{I} - \mathbf{A})^{-1}$ is equivalent to the *Leontief inverse* or the *total requirements* matrix which appears in input-output analyses of national economies.[3]

Finally, the assumption of equal distribution of consumption and exports is used to derive the matrix

$$\overline{\mathbf{R}} = \hat{\mathbf{c}} \cdot \mathbf{R}$$

for which $\hat{\mathbf{c}}$ is vector comprised of elements $c_i = \frac{1}{x_i}(x_i - \sum_k z_{kj})$, where x_i is the DMI in country i, and z_{kj} the exports of country k to country j.

For example, if for a given commodity the in-country production is 1800 tonnes, the imports 50 tonnes, and the exports are 500 tonnes, the consumption is equal to 1800+50-500 = 1350, yielding a consumption share of 1350/1850. Kastner and colleagues also provide the example of a hypothetical four-country trading system to demonstrate the impact of the correction (SI Table 2).

Following trade correction, we aggregate the commodity level detail to food groups relevant for health analyses, including to fruits, vegetables, pulses, nuts, and red meat (SI Table 3).

SI Table 1. Complete list of commodity equivalents used for converting from secondary products to primary equivalents. Those given a conversion factor of 0, and a primary value of "out" are excluded.

Item	FAO Item Code	Conversion factor	Primary
Agave fibres nes	800	0	out
Alfalfa meal and pellets	862	0	out
Almonds shelled	231	2.5	almond
Almonds, with shell	221	1	almond
Animals live nes	1171	0	out
Animals, live, non-food	1169	0	out
Anise, badian, fennel, coriander	711	1	aniseetc
Apples	515	1	apple
Apricots	526	1	apricot
Apricots, dry	527	3.448275862	apricot
Areca nuts	226	0	areca
Artichokes	366	1	artichoke
Asparagus	367	1	asparagus
Asses	1107	0	out
Avocados	572	1	avocado
Bacon and ham	1039	1.298701299	pig
Bambara beans	203	1	bambara
Bananas	486	1	banana
Barley	44	1	barley
Barley, pearled	46	0.149510354	barley
Bastfibres, other	782	0	out
Beans, dry	176	1	bean
Beans, green	414	1	greenbean
Beef and Buffalo Meat	1806	1	out
Beehives	1181	0	out
Beer of barley	51	0.18637592	barley
Beeswax	1183	0	out
Beet pulp	169	1	sugarbeet
Berries nes	558	0	berrynes
Beverages, distilled alcoholic	634	0	out
Beverages, fermented rice	39	0.476190476	rice
Beverages, non alcoholic	633	0	out
Blueberries	552	1	blueberry
Bran, buckwheat	91	1.052631579	buckwheat
Bran, maize	59	1.01010101	maize
Bran, millet	81	1.041666667	millet
Bran, sorghum	85	1.020408163	sorghum
Bran, wheat	17	1.01010101	wheat
Brazil nuts, shelled	229	2.272727273	brazil
Brazil nuts, with shell	216	1	brazil
Bread	20	0.878348704	wheat
Broad beans, horse beans, dry	181	1	broadbean

Buckwheat	89	1	buckwheat
Buffaloes	946	0	out
Bulgur	21	1.052631579	wheat
Butter of karite nuts	264	3.846153846	out
Butter, cow milk	886	1.020408163	cowmilk
Buttermilk, curdled, acidified milk	893	1.020408163	cowmilk
Cabbages and other brassicas	358	1	cabbage
Cake, copra	253	4.909180167	coconut
Cake, cottonseed	332	1.287001287	cotton
Cake, groundnuts	245	1.47275405	groundnut
Cake, hempseed	338	1.086956522	hempseed
Cake, kapok	314	1.515151515	kapok
Cake, linseed	335	1.020408163	linseed
Cake, maize	61	1.041341248	maize
Cake, mustard	294	1.041666667	mustard
Cake, palm kernel	259	4.081632653	oilpalm
Cake, rapeseed	272	1.020408163	rapeseed
Cake, rice bran	37	1.709401709	rice
Cake, safflower	282	1.086956522	safflower
Cake, sesame seed	291	1.063829787	sesame
Cake, soybeans	238	1.030927835	soybean
Cake, sunflower	269	1.136363636	sunflower
Camelids, other	1157	0	out
Camels	1126	0	out
Canary seed	101	1	canaryseed
Cane tops	630	0	out
Carobs	461	0	carob
Carrots and turnips	426	1	carrot
Cashew nuts, shelled	230	4.166666667	cashew
Cashew nuts, with shell	217	1	cashew
Cashewapple	591	1	cashewapple
Cassava	125	1	cassava
Cassava dried	128	3.571428571	cassava
Cassava leaves	378	0	out
Castor oil seed	265	1	castor
Cattle	866	0	out
Cauliflowers and broccoli	393	1	cauliflower
Cereal preparations nes	113	0	out
Cereals nes	108	1	cerealnes
Cereals, breakfast	41	1.176470588	wheat
Cereals, Total	1717	0	out
Cheese, processed	907	5.649717514	cowmilk
Cheese, sheep milk	984	4.615384615	sheepmilk
Cheese, whole cow milk	901	1.136363636	cowmilk
Cherries	531	1	cherry
Cherries, sour	530	1	sourcherry

Chestnut	220	1	chestnut
Chick peas	191	1	chickpea
Chickens	1057	0	out
Chicory roots	459	0	chicory
Chillies and peppers, dry	689	1	pimento
Chillies and peppers, green	401	1	chilleetc
Chocolate products nes	666	0.212765957	cocoa
Cider etc	517	0	out
Cigarettes	828	0	out
Cigars, cheroots	829	0	out
Cinnamon (cannella)	693	1	cinnamon
Citrus Fruit, Total	1804	0	out
Cloves	698	1	clove
Cocoa, beans	661	1	cocoa
Cocoa, butter	664	1	cocoa
Cocoa, paste	662	1	cocoa
Cocoa, powder & cake	665	1	cocoa
Coconuts	249	1	coconut
Coconuts, desiccated	250	5	coconut
Cocoons, unreelable & waste	1187	0	out
Coffee, extracts	659	2.941176471	coffee
Coffee, green	656	1	coffee
Coffee, husks and skins	660	0	out
Coffee, roasted	657	1.25	coffee
Coffee, substitutes containing coffee	658	1.428571429	coffee
Coir	813	0	coir
Copra	251	4.761904762	coconut
Cotton lint	767	0.965250965	cotton
Cotton linter	770	1.287001287	cotton
Cotton waste	769	0.965250965	cotton
Cotton, carded, combed	768	0	out
Cottonseed	329	0.965250965	cotton
Cow peas, dry	195	0	cowpea
Cranberries	554	1	cranberry
Cream fresh	885	6.666666667	cowmilk
Crude materials	1293	0	out
Cucumbers and gherkins	397	1	cucumberetc
Currants	550	1	currant
Dates	577	1	date
Dregs from brewing, distillation	654	0	out
Ducks	1068	0	out
Eggplants (aubergines)	399	1	eggplant
Eggs Primary	1783	1	egg
Eggs, dried	1064	3.555555556	egg
Eggs, hen, in shell	1062	1	egg
Eggs, hen, in shell (number)	1067	0	out

Eggs, liquid	1063	1	egg
Eggs, other bird, in shell	1091	1	egg
Eggs, other bird, in shell (number)	1092	0	out
Fat nes, prepared	1243	0	out
Fat, buffaloes	949	0	out
Fat, camels	1129	0	out
Fat, cattle	869	0	out
Fat, goats	1019	0	out
Fat, liver prepared (foie gras)	1060	0	out
Fat, pigs	1037	0	out
Fat, sheep	979	0	out
Fatty acids	1276	0	out
Fatty substance residues	1277	0	out
Feed and meal, gluten	846	0	out
Feed minerals	855	0	out
Feed supplements	850	0	out
Feed, compound nes	845	0	out
Feed, pulp of fruit	628	0	out
Feed, vegetable products nes	652	0	out
Fibre crops nes	821	0	out
Fibre Crops Primary	1753	0	out
Figs	569	1	fig
Figs dried	570	2.857142857	fig
Flax fibre and tow	773	1	flax
Flax fibre raw	771	0	out
Flax tow waste	774	0	out
Flour, cassava	126	4.166666667	cassava
Flour, cereals	111	1.101591187	out
Flour, maize	58	1.01010101	maize
Flour, mixed grain	104	1.041666667	out
Flour, mustard	295	1.5625	mustard
Flour, potatoes	117	5	potato
Flour, pulses	212	1.052631579	pulsenes
Flour, rice	38	1	rice
Flour, roots and tubers nes	150	2.272727273	rootnes
Flour, wheat	16	1.01010101	wheat
Fonio	94	1	fonio
Food prep nes	1232	0	out
Food preparations, flour, malt extract	115	0	out
Food wastes	653	0	out
Forage products	651	1	out
Fructose and syrup, other	166	0	out
Fruit Primary	1738	0	out
Fruit, citrus nes	512	1	citrusnes
Fruit, cooked, homogenized preparations	626	1	out
Fruit, dried nes	620	1	out

Fruit, fresh nes	619	1	fruitnes
Fruit, pome nes	542	0	out
Fruit, prepared nes	623	1	out
Fruit, stone nes	541	0	out
Fruit, tropical fresh nes	603	1	tropicalnes
Garlic	406	1	garlic
Germ, maize	57	1.01010101	maize
Ghee, buffalo milk	953	6.538461538	buffalomilk
Ginger	720	1	ginger
Glucose and dextrose	172	0	out
Goats	1016	0	out
Gooseberries	549	1	gooseberry
Grain, mixed	103	1	out
Grapefruit (inc. pomelos)	507	1	grapefruitetc
Grapes	560	1	grape
Grease incl. lanolin wool	994	0	out
Groundnuts, prepared	246	0	out
Groundnuts, shelled	243	1.428571429	groundnut
Groundnuts, with shell	242	1	groundnut
Gums, natural	839	1	gums
Hair, fine	1218	0	out
Hair, goat, coarse	1031	0	out
Hay (clover, lucerne,etc)	858	0	out
Hay (unspecified)	859	0	out
Hazelnuts, shelled	233	2.5	hazelnut
Hazelnuts, with shell	225	1	hazelnut
Hemp tow waste	777	0	out
Hempseed	336	1	hempseed
Hides and skins nes, fresh	1213	0	out
Hides nes	1216	0	out
Hides, buffalo, dry salted	959	0	out
Hides, buffalo, fresh	957	0	out
Hides, buffalo, wet salted	958	0	out
Hides, camel, wet salted	1134	0	out
Hides, cattle, fresh	919	0	out
Hides, cattle, wet salted	920	0	out
Hides, horse, dry salted	1104	0	out
Honey, natural	1182	0	out
Hops	677	1	hop
Horses	1096	0	out
Ice cream and edible ice	910	2	cowmilk
Infant food	109	0	out
Jojoba seed	277	0	out
Juice, apple, concentrated	519	0	apple
Juice, apple, single strength	518	0	apple
Juice, citrus, concentrated	514	1.897533207	citrusnes

Juice, citrus, single strength	513	1.897533207	citrusnes
Juice, fruit nes	622	0	fruitnes
Juice, grape	562	1.333333333	grape
Juice, grapefruit	509	1.639344262	grapefruitetc
Juice, grapefruit, concentrated	510	1.639344262	grapefruitetc
Juice, lemon, concentrated	499	2.057613169	lemonlime
Juice, lemon, single strength	498	0	lemonlime
Juice, orange, concentrated	492	10	orange
Juice, orange, single strength	491	2.272727273	orange
Juice, pineapple	576	1.388888889	pineapple
Juice, pineapple, concentrated	580	5	pineapple
Juice, plum, concentrated	539	12.5	plum
Juice, tomato	390	1.5625	tomato
Jute	780	1	out
Kapok fibre	778	3.03030303	out
Kapok fruit	310	1	kapok
Kapokseed in shell	311	1.515151515	kapok
Karite nuts (sheanuts)	263	1	karite
Kiwi fruit	592	1	kiwi
Kola nuts	224	1	kolanut
Lactose	173	1.020408163	cowmilk
Lard	1043	0	out
Leeks, other alliaceous vegetables	407	0	out
Lemons and limes	497	1	lemonlime
Lentils	201	1	lentil
Lettuce and chicory	372	1	lettuce
Linseed	333	1	linseed
Lupins	210	1	lupin
Macaroni	18	1.01010101	wheat
Maize	56	1	maize
Maize, green	446	1	greencorn
Malt	49	1.369863014	barley
Mangoes, mangosteens, guavas	571	1	mango
Manila fibre (abaca)	809	1	out
Maple sugar and syrups	160	0	out
Margarine, liquid	1241	0	out
Margarine, short	1242	0	out
Mat<e9>	671	1	mate
Maté	671	1	mate
Meal, meat	1173	0	out
Meat nes	1166	0	out
Meat, ass	1108	0	out
Meat, beef and veal sausages	874	1	cowmeat
Meat, beef, preparations	875	1	cowmeat
Meat, bird nes	1089	0	out
Meat, buffalo	947	1	buffalomeat

Meat, camel	1127	0	out
Meat, cattle	867	1	cowmeat
Meat, cattle, boneless (beef & veal)	870	1.408450704	cowmeat
Meat, chicken	1058	1	chickenmeat
Meat, chicken, canned	1061	1.111111111	chickenmeat
Meat, dried nes	1164	0	out
Meat, duck	1069	1	duckmeat
Meat, game	1163	0	out
Meat, goat	1017	1	goatmeat
Meat, goose and guinea fowl	1073	1	goosemeat
Meat, horse	1097	0	out
Meat, mule	1111	0	out
Meat, other camelids	1158	0	out
Meat, other rodents	1151	0	out
Meat, pig	1035	1	pig
Meat, pig sausages	1041	1.176470588	pig
Meat, pig, preparations	1042	1.136363636	pig
Meat, pork	1038	1.369863014	pig
Meat, Poultry	1808	1	out
Meat, rabbit	1141	0	out
Meat, sheep	977	1	sheepmeat
Meat, Total	1765	0	out
Meat, turkey	1080	1	turkeymeat
Melons, other (inc.cantaloupes)	568	1	melonseed
Melonseed	299	1	melonseed
Milk, products of natural constituents nes	909	2	cowmilk
Milk, skimmed cow	888	1.020408163	cowmilk
Milk, skimmed dried	898	10.20408163	cowmilk
Milk, Total	1780	0	out
Milk, whole condensed	889	3.03030303	cowmilk
Milk, whole dried	897	6.666666667	cowmilk
Milk, whole evaporated	894	2.5	cowmilk
Milk, whole fresh buffalo	951	1	buffalomilk
Milk, whole fresh camel	1130	0	out
Milk, whole fresh cow	882	1	cowmilk
Milk, whole fresh goat	1020	1	goatmilk
Milk, whole fresh sheep	982	1	sheepmilk
Millet	79	1	millet
Mixes and doughs	114	0	out
Molasses	165	1	sugarcane
Mules	1110	0	out
Mushrooms and truffles	449	1	mushroom
Mushrooms, canned	451	1.136363636	mushroom
Mustard seed	292	1	mustard
Nutmeg, mace and cardamoms	702	1	nutmeg
Nuts nes	234	1	nutnes

Nuts, prepared (exc. groundnuts)	235	2.083333333	nutnes
Oats	75	1	oats
Oats rolled	76	1.369863014	oats
Offals, edible, buffaloes	948	0	out
Offals, edible, camels	1128	0	out
Offals, edible, cattle	868	0	out
Offals, edible, goats	1018	0	out
Offals, horses	1098	0	out
Offals, liver chicken	1059	0	out
Offals, liver duck	1075	0	out
Offals, liver geese	1074	0	out
Offals, other camelids	1159	0	out
Offals, pigs, edible	1036	0	out
Offals, sheep,edible	978	0	out
Oil palm fruit	254	1	oilpalm
Oil, boiled etc	1274	0	out
Oil, castor beans	266	2.5	castor
Oil, citronella	737	0	out
Oil, coconut (copra)	252	4.909180167	coconut
Oil, cottonseed	331	1.287001287	cotton
Oil, essential nes	753	0	out
Oil, groundnut	244	1.47275405	groundnut
Oil, kapok	313	1.515151515	kapok
Oil, linseed	334	1.020408163	linseed
Oil, maize	60	1.041341248	maize
Oil, olive residues	274	1.923076923	olive
Oil, olive, virgin	261	1.923076923	olive
Oil, palm	257	4	oilpalm
Oil, palm kernel	258	4.081632653	oilpalm
Oil, poppy	297	1.086956522	poppy
Oil, rapeseed	271	1.020408163	rapeseed
Oil, rice bran	36	1.709401709	rice
Oil, safflower	281	1.086956522	safflower
Oil, sesame	290	1.063829787	sesame
Oil, soybean	237	1.030927835	soybean
Oil, sunflower	268	1.136363636	sunflower
Oil, vegetable origin nes	340	1.086956522	out
Oilcrops	1731	0	out
Oilcrops, Cake Equivalent	1841	0	out
Oilcrops, Oil Equivalent	1732	0	out
Oils, fats of animal nes	1168	0	out
Oilseeds nes	339	1	oilseednes
Okra	430	1	okra
Olives	260	1	olive
Olives preserved	262	1.25	olive
Onions, dry	403	1	onion

Onions, shallots, green	402	1	onion
Oranges	490	1	orange
Papayas	600	1	papaya
Pastry	22	0.878348704	wheat
Peaches and nectarines	534	1	peachetc
Peanut butter	247	2.100840336	groundnut
Pears	521	1	pear
Peas, dry	187	1	pea
Peas, green	417	1.23985	greenpea
Pepper (piper spp.)	687	1	pepper
Peppermint	748	1	peppermint
Persimmons	587	1	persimmon
Pet food	843	0	out
Pigeon peas	197	1	pigeonpea
Pigeons, other birds	1083	0	out
Pigs	1034	0	out
Pineapples	574	1	pineapple
Pineapples canned	575	1.388888889	pineapple
Pistachios	223	1	pistachio
Plantains and others	489	1	plantain
Plums and sloes	536	1	plum
Plums dried (prunes)	537	2.857142857	plum
Poppy seed	296	1	poppy
Potato offals	120	1.020408163	potato
Potatoes	116	1	potato
Potatoes, frozen	118	1.020408163	potato
Pulses nes	211	1	pulsenes
Pulses, Total	1726	1	out
Pumpkins, squash and gourds	394	1	pumpkinetc
Pyrethrum, dried	754	1	pyrethrum
Pyrethrum, extraction	755	0	pyrethrum
Quinces	523	1	quince
Quinoa	92	1	quinoa
Rabbits and hares	1140	0	out
Raisins	561	4	grape
Ramie	788	0	ramie
Rapeseed	270	1	rapeseed
Raspberries	547	1	rasberry
Rice, broken	32	1.666666667	rice
Rice, husked	28	1.298701299	rice
Rice, milled	31	1.333333333	rice
Rice, milled/husked	29	0	out
Rice, paddy	27	1	rice
Rice, paddy (rice milled equivalent)	30	1.492537313	out
Rodents, other	1150	0	out
Roots and tubers nes	149	1	rootnes

Roots and Tubers, Total	1720	0	out
Rubber natural dry	837	0	out
Rubber, natural	836	1	out
Rye	71	1	rye
Safflower seed	280	1	safflower
Seed cotton	328	1	cotton
Sesame seed	289	1	sesame
Sheep	976	0	out
Sheep and Goat Meat	1807	1	out
Silk, raw	1186	0	out
Silk-worm cocoons, reelable	1185	0	out
Sisal	789	0	out
Skins, calve, wet salted	928	0	out
Skins, goat, fresh	1025	0	out
Skins, goat, wet salted	1026	0	out
Skins, sheep, dry salted	997	0	out
Skins, sheep, fresh	995	0	out
Skins, sheep, wet salted	996	0	out
Skins, sheep, with wool	999	0	out
Snails, not sea	1176	0	out
Sorghum	83	1	sorghum
Soya paste	240	0.222222222	soybean
Soya sauce	239	0.285714286	soybean
Soybeans	236	1	soybean
Spices nes	723	1	spicenes
Spinach	373	1	spinach
Starch, cassava	129	5	cassava
Straw husks	635	0	out
Strawberries	544	1	strawberry
String beans	423	1	stringbean
Sugar beet	157	0.84	sugarbeet
Sugar cane	156	1	sugarcane
Sugar confectionery	168	0.5	sugarnes
Sugar crops nes	161	1	sugarnes
Sugar Crops Primary	1723	0	out
Sugar flavoured	171	0.5	sugarnes
Sugar nes	167	1	sugarnes
Sugar non-centrifugal	163	1	sugarnes
Sugar Raw Centrifugal	162	11.11111111	sugarnes
Sugar refined	164	2.409638554	sugarnes
Sunflower seed	267	1	sunflower
Sweet corn frozen	447	3.703703704	greencorn
Sweet corn prep or preserved	448	2.5	greencorn
Sweet potatoes	122	1	sweetpotato
Tallow	1225	0	out
Tallowtree seed	305	0	out

Tangerines, mandarins, clementines, satsumas	495	1	tangetc
Taro (cocoyam)	136	1	taro
Tea	667	1	tea
Tea, mate extracts	672	1	tea
Tobacco products nes	831	0	out
Tobacco, unmanufactured	826	1	out
Tomatoes	388	1	tomato
Tomatoes, paste	391	5	tomato
Tomatoes, peeled	392	1.5625	tomato
Treenuts, Total	1729	0	out
Triticale	97	1	triticale
Tung nuts	275	1	tung
Turkeys	1079	0	out
Turnips for fodder	646	1	out
Vanilla	692	1	vanilla
Vegetable tallow	306	0	out
Vegetables in vinegar	471	1.25	vegetablenes
Vegetables Primary	1735	0	out
Vegetables, canned nes	465	1.111111111	vegetablenes
Vegetables, dehydrated	469	6.666666667	vegetablenes
Vegetables, dried nes	464	5	vegetablenes
Vegetables, fresh nes	463	1	vegetablenes
Vegetables, fresh or dried products nes	460	0	out
Vegetables, frozen	473	1.25	vegetablenes
Vegetables, homogenized preparations	476	1	out
Vegetables, leguminous nes	420	1	greenbroadbean
Vegetables, preserved nes	472	1.25	vegetablenes
Vegetables, preserved, frozen	475	1.111111111	vegetablenes
Vegetables, temporarily preserved	474	1.111111111	vegetablenes
Vermouths & similar	565	0	out
Vetches	205	1	vetch
Vitamins	853	0	out
Wafers	110	0.878348704	wheat
Walnuts, shelled	232	2.358490566	walnut
Walnuts, with shell	222	1	walnut
Watermelons	567	1	watermelon
Waters,ice etc	631	0	out
Waxes vegetable	1296	0	out
Wheat	15	1	wheat
Whey, condensed	890	1.020408163	cowmilk
Whey, dry	900	1.020408163	cowmilk
Wine	564	1.111111111	out
Wool, degreased	988	0	out
Wool, greasy	987	0	out
Wool, hair waste	1009	0	out
Yams	137	1	yam

Yautia (cocoyam)	135	1	yautia
Yoghurt	891	1.020408163	cowmilk
Yoghurt, concentrated or not	892	1.020408163	cowmilk

SI Table 2 Example of the impact of the trade matrix correction, adapted from Kastner and colleagues.[2]

Total apparent consumption	Place of consumption	Reported trade links $\mathbf{R}^A$				After correction $\bar{\mathbf{R}}$			
		A	B	C	D	A	B	C	D
400	A	160	0	80	160	174	191	31	4
450	B	0	450	0	0	0	450	0	0
250	C	23	159	45	23	11	188	49	1
210	D	23	91	91	5	14	171	20	5

SI Table 3. Classification of food commodities, with names according to Carlson and colleagues.[4]

Fruits	Vegetables	Pulses	Nuts	Red meat
apple	artichoke	bambara	almond	cowmeat
apricot	asparagus	bean	brazil	pig
avocado	greenbean	broadbean	cashew	sheepmeat
banana	cabbage	chickpea	chestnut	goatmeat
berrynes	carrot	cowpea	hazelnut	
blueberry	cauliflower	lentil	nutnes	
carob	chilleetc	lupin	pistachio	
cashewapple	cucumberetc	pea	walnut	
cherry	eggplant	pigeonpea	groundnut	
citrusnes	garlic	pulsenes		
cranberry	greenbroadbean	vetch		
currant	lettuce			
date	greencorn			
fig	mushroom			
fruitnes	okra			
tropicalnes	greenonion			
gooseberry	onion			
grapefruitetc	greenpea			
grape	pumpkinetc			
kiwi	spinach			
lemonlime	stringbean			
mango	tomato			
orange	vegetablenes			
papaya				
peachetc				
pear				
persimmon				
pineapple				
plantain				
plum				
quince				
rasberry				
sourcherry				
stonefruitnes				

strawberry
tangetc
watermelon
melonetc

Comparative risk assessment

We estimated the mortality and disease burden attributable to dietary risk factors by calculating population impact fractions (PIFs) which represent the proportions of disease cases that would be avoided when the risk exposure was changed from a baseline situation to a counterfactual situation. For calculating PIFs, we used the general formula[5–7]:

$$PIF = \frac{\int RR(x)P(x)dx - \int RR(x)P'(x)dx}{\int RR(x)P(x)dx}$$

where $RR(x)$ is the relative risk of disease for risk factor level x, $P(x)$ is the number of people in the population with risk factor level x in the baseline scenario, and $P'(x)$ is the number of people in the population with risk factor level x in the counterfactual scenario. We assumed that changes in relative risks follow a dose-response relationship,[6] and that PIFs combine multiplicatively, i.e. $PIF = 1 - \prod_i (1 - PIF_i)$ where the *i*'s denote independent risk factors.[6,8]

The number of avoided deaths due to the change in risk exposure of risk *i*, $\Delta deaths_i$, was calculated by multiplying the associated PIF by disease-specific death rates, *DR*, and by the number of people alive within a population, *P*:

$$\Delta deaths_i(r, a, d) = PIF_i(r, d) \cdot DR(r, a, d) \cdot P(r, a)$$

where PIFs are differentiated by region *r* and disease/cause of death *d*; the death rates are differentiated by region, age group *a*, and disease; the population groups are differentiated by region and age group; and the change in the number of deaths is differentiated by region, age group and disease.

We used publicly available data sources to parameterize the comparative risk analysis. Mortality and population data were adopted from the Global Burden of Disease project.[9] The relative risk estimates that relate the risk factors to the disease endpoints were adopted from meta-analyses of prospective cohort studies for dietary risks.[10–15] As our analysis was primarily focused on mortality from chronic diseases, we focused on adults aged 20 year or older, and we adjusted the relative-risk estimates for attenuation with age based on a pooled analysis of cohort studies focussed on metabolic risk factors,[16] in line with other assessments.[7,17]

SI Table 4 provides an overview of the relative-risk parameters used. The selection of risk-disease associations used in the health analysis was supported by available criteria used to judge the certainty of evidence, such as the Bradford-Hill criteria used by the Nutrition and Chronic Diseases Expert Group (NutriCoDE),[17] the World-Cancer-Research-Fund criteria used by the Global Burden of Disease project,[18] as well as NutriGrade (SI Table 5).[19] The certainty of evidence supporting the associations of dietary risks and disease outcomes as

used here were graded as moderate or high with NutriGrade,[13–15] and/or assessed as probable or convincing by the Nutrition and Chronic Diseases Expert Group,[17] and by the World Cancer Research Fund.[20] The certainty of evidence grading in each case relates to the general relationship between a risk factor and a health outcome, and not to a specific relative-risk value.

We did not include all available risk-disease associations that were graded as having a moderate certainty of evidence and showed statistically significant results in the meta-analyses that included NutriGrade assessments.[13–15] That was because for some associations, such as for milk and fish, more detailed meta-analyses (with more sensitivity analyses) were available that indicated potential confounding with other major dietary risks or health status at baseline.[21–23] Such sensitivity analyses were not presented in the meta-analyses that included NutriGrade assessments, but they are important for health assessments that evaluate changes in multiple risk factors.

We calculated uncertainty intervals associated with changes in mortality based on standard methods of error propagation and the confidence intervals of the relative risk parameters. For the error propagation, we approximated the error distribution of the relative risks by a normal distribution and used that side of deviations from the mean which was largest. This method leads to conservative and potentially larger uncertainty intervals as probabilistic methods, such as Monte Carlo sampling, but it has significant computational advantages, and is justified for the magnitude of errors dealt with here (<50%) (see e.g. IPCC Uncertainty Guidelines).

SI Table 4. Relative risk parameters (mean and low and high values of 95% confidence intervals) for dietary risks.

Food group	Endpoint	Unit	RR mean	RR low	RR high	Reference
Red meat	CHD	100 g/d	1.15	1.08	1.23	Bechthold et al (2019)
	Stroke	100 g/d	1.12	1.06	1.17	Bechthold et al (2019)
	Colorectal cancer	100 g/d	1.12	1.06	1.19	Schwingshackl et al (2018)
	Type 2 diabetes	100 g/d	1.17	1.08	1.26	Schwingshackl et al (2017)
Fruits	CHD	100 g/d	0.95	0.92	0.99	Aune et al (2017)
	Stroke	100 g/d	0.77	0.70	0.84	Aune et al (2017)
	Cancer	100 g/d	0.94	0.91	0.97	Aune et al (2017)
Vegetables	CHD	100 g/d	0.84	0.80	0.88	Aune et al (2017)
	Cancer	100 g/d	0.93	0.91	0.95	Aune et al (2017)
Legumes	CHD	57 g/d	0.86	0.78	0.94	Afshin et al (2014)
Nuts	CHD	28 g/d	0.71	0.63	0.80	Aune et al (2016)

SI Table 5. Overview of existing ratings on the certainty of evidence for a statistically significant association between a risk factor and a disease endpoint. The ratings include those of the Nutrition and Chronic Diseases Expert Group (NutriCoDE),[17] the World Cancer Research Fund,[20] and NutriGrade.[13–15] The ratings relate to the risk-disease associations in general, and not to the specific relative-risk factor used for those associations in this analysis.

Food group	Endpoint	Association	Certainty of evidence
Fruits	CHD	reduction	NutriCoDE: probable or convincing;<br>NutriGrade: moderate quality of meta-evidence
	Stroke	reduction	NutriCoDE: probable or convincing<br>NutriGrade: moderate quality of meta-evidence
	Cancer	reduction	WCRF: strong evidence (probable) for some cancers<br>NutriGrade: moderate quality of meta-evidence for colorectal cancer
Vegetables	CHD	reduction	NutriCoDE: probable or convincing<br>NutriGrade: moderate quality of meta-evidence
	Cancer	reduction	WCRF: strong evidence (probable) for non-starchy vegetables and some cancers<br>NutriGrade: moderate quality of meta-evidence for colorectal cancer
Legumes	CHD	reduction	NutriCoDE: probable or convincing<br>NutriGrade: moderate quality of meta-evidence
Nuts	CHD	reduction	NutriCoDE: probable or convincing<br>NutriGrade: moderate quality of meta-evidence
Red meat	CHD	increase	NutriGrade: moderate quality of meta-evidence
	Stroke	increase	NutriGrade: moderate quality of meta-evidence
	Cancer	increase	WCRF: strong evidence (probable) for colorectal cancer<br>NutriGrade: moderate quality of meta-evidence for colorectal cancer
	Type-2 diabetes	increase	NutriCoDE: probable or convincing<br>NutriGrade: high quality of meta-evidence

NutriCoDE: Nutrition and Chronic Diseases Expert Group

NutriGrade: Grading of Recommendations Assessment, Dvelopment, and Evaluation (GRADE) tailored to nutrition research

WCRF: World Cancer Research Fund

Supplementary results

SI Table 6. Regional flows of international food trade between exporting and importing regions (in kilo tonnes per year, kt/yr, and as a percentage of production in the exporting region, %prod).

Food group	Exporter	Importer						
		World (%prod)	World (kt/yr)	Africa (kt/yr)	Americas (kt/yr)	Asia (kt/yr)	Europe (kt/yr)	Oceania (kt/yr)
fruits	World	11	86,128	2,102	23,277	20,715	39,577	457
	Africa	6	7,465	905	369	2,324	3,857	10
	Americas	27	42,364	407	21,067	4,186	16,542	161
	Asia	5	19,936	329	1,343	12,406	5,669	188
	Europe	18	14,982	455	401	924	13,188	14
	Oceania	18	1,381	5	97	874	321	84
vegetables	World	5	57,784	3,121	11,813	16,404	25,715	731
	Africa	4	3,697	644	194	721	2,110	28
	Americas	13	11,497	77	9,274	1,264	774	108
	Asia	2	22,079	1,457	1,737	12,660	5,931	293
	Europe	21	19,934	933	603	1,395	16,789	214
	Oceania	15	578	10	5	363	111	88
legumes	World	3	12,381	946	1,526	7,740	2,142	27
	Africa	2	522	180	4	281	56	0
	Americas	3	7,789	316	1,375	5,424	658	15
	Asia	2	1,561	82	123	1,162	191	4
	Europe	10	2,222	252	14	727	1,229	0
	Oceania	15	287	116	10	146	8	8
nuts	World	12	8,430	381	954	3,830	3,179	86
	Africa	7	1,480	159	35	1,164	120	2
	Americas	48	4,265	153	765	1,259	2,036	52
	Asia	5	2,108	62	135	1,214	673	24
	Europe	30	398	5	11	75	306	1
	Oceania	63	179	2	7	118	44	7
red meat	World	11	25,460	627	4,131	10,900	9,415	387
	Africa	1	116	39	0	56	21	0
	Americas	15	9,732	375	3,240	5,282	657	178
	Asia	1	744	53	1	652	35	3
	Europe	25	11,737	148	219	2,640	8,564	165
	Oceania	52	3,131	13	671	2,269	137	42

SI Table 7. Changes in regional food availability per person (in grams per person per day, g/d, and as a percentage of regional food demand) in importing regions due to foods exported from exporting regions.

Food group	Exporter	Unit	Importer					
			World	Africa	Americas	Asia	Europe	Oceania
fruits	World	(%dmnd)	14	2	22	6	64	10
	World	(g/d)	31	4	63	12	145	30
	Africa	(g/d)	3	2	1	1	14	1
	Americas	(g/d)	15	1	57	2	60	11
	Asia	(g/d)	7	1	4	7	21	12
	Europe	(g/d)	5	1	1	1	48	1
	Oceania	(g/d)	0	0	0	1	1	6
vegetables	World	(%dmnd)	5	4	16	2	32	22
	World	(g/d)	21	7	32	10	94	48
	Africa	(g/d)	1	1	1	0	8	2
	Americas	(g/d)	4	0	25	1	3	7
	Asia	(g/d)	8	3	5	8	22	19
	Europe	(g/d)	7	2	2	1	61	14
	Oceania	(g/d)	0	0	0	0	0	6
legumes	World	(%dmnd)	19	7	19	20	100	29
	World	(g/d)	4	2	4	5	8	2
	Africa	(g/d)	0	0	0	0	0	0
	Americas	(g/d)	3	1	4	3	2	1
	Asia	(g/d)	1	0	0	1	1	0
	Europe	(g/d)	1	1	0	0	4	0
	Oceania	(g/d)	0	0	0	0	0	1
nuts	World	(%dmnd)	21	5	24	15	97	35
	World	(g/d)	3	1	3	2	12	6
	Africa	(g/d)	1	0	0	1	0	0
	Americas	(g/d)	2	0	2	1	7	3
	Asia	(g/d)	1	0	0	1	2	2
	Europe	(g/d)	0	0	0	0	1	0
	Oceania	(g/d)	0	0	0	0	0	0
red meat	World	(%dmnd)	11	4	8	10	23	14
	World	(g/d)	9	1	11	7	34	25
	Africa	(g/d)	0	0	0	0	0	0
	Americas	(g/d)	3	1	9	3	2	12
	Asia	(g/d)	0	0	0	0	0	0
	Europe	(g/d)	4	0	1	2	31	11
	Oceania	(g/d)	1	0	2	1	1	3

SI Table 8. Changes in mortality attributed to foods that are related to dietary risks and that are traded from exporting to importing regions, measured in changes in the number of deaths (deaths) and as a percentage of the diet-related burden of the same set of risk factors (%burden).

Dietary risks	Exporter	Unit	Importer					
			World	Africa	Americas	Asia	Europe	Oceania
all risks	World	%burden	19	6	23	9	45	18
	World	deaths	-1,225,880	-32,562	-209,375	-301,398	-675,360	-7,186
	Africa	deaths	-117,795	-7,789	-4,537	-49,349	-55,877	-243
	Americas	deaths	-507,032	-7,669	-179,440	-79,381	-238,211	-2,331
	Asia	deaths	-364,715	-7,800	-23,274	-166,914	-163,652	-3,076
	Europe	deaths	-231,310	-7,841	-4,763	-4,856	-213,323	-526
	Oceania	deaths	-5,030	-1,463	2,640	-899	-4,298	-1,010
fruits	World	%burden	30	4	76	10	82	23
	World	deaths	-597,038	-8,889	-118,889	-126,217	-340,821	-2,222
	Africa	deaths	-48,112	-3,517	-1,893	-9,380	-33,273	-50
	Americas	deaths	-271,468	-1,851	-107,533	-27,142	-134,136	-808
	Asia	deaths	-150,378	-1,464	-7,030	-79,512	-61,488	-884
	Europe	deaths	-117,768	-2,035	-1,941	-4,014	-109,713	-65
	Oceania	deaths	-9,312	-23	-493	-6,169	-2,212	-415
vegetables	World	%burden	24	5	21	10	57	26
	World	deaths	-380,217	-9,301	-72,447	-66,648	-227,991	-3,830
	Africa	deaths	-22,248	-1,812	-1,225	-2,364	-16,703	-144
	Americas	deaths	-70,364	-303	-57,276	-5,932	-6,261	-591
	Asia	deaths	-130,088	-3,862	-10,528	-50,419	-63,764	-1,516
	Europe	deaths	-154,778	-3,295	-3,391	-6,513	-140,472	-1,107
	Oceania	deaths	-2,739	-30	-27	-1,419	-791	-473
legumes	World	%burden	6	6	5	6	5	3
	World	deaths	-98,163	-8,286	-11,584	-55,606	-22,439	-247
	Africa	deaths	-3,511	-651	-37	-1,907	-915	-1
	Americas	deaths	-58,809	-2,588	-10,127	-39,373	-6,599	-121
	Asia	deaths	-12,609	-758	-1,166	-8,189	-2,466	-30
	Europe	deaths	-20,672	-2,861	-148	-5,272	-12,387	-3
	Oceania	deaths	-2,562	-1,429	-104	-865	-72	-91
nuts	World	%burden	35	11	25	28	56	83
	World	deaths	-299,852	-9,082	-26,168	-106,590	-155,510	-2,502
	Africa	deaths	-44,354	-1,975	-1,383	-35,831	-5,115	-49
	Americas	deaths	-156,274	-4,872	-19,543	-32,707	-97,640	-1,512
	Asia	deaths	-75,543	-2,002	-4,575	-31,701	-36,595	-669
	Europe	deaths	-17,507	-169	-393	-2,722	-14,203	-19
	Oceania	deaths	-6,175	-64	-273	-3,629	-1,957	-252
red meat	World	%burden	14	10	9	10	24	15
	World	deaths	147,160	2,959	18,754	53,295	70,546	1,607
	Africa	deaths	385	151	0	122	110	0
	Americas	deaths	48,783	1,935	14,102	25,820	6,225	701
	Asia	deaths	3,206	275	3	2,496	413	19
	Europe	deaths	79,021	515	1,110	13,666	63,063	667
	Oceania	deaths	15,766	82	3,539	11,191	734	220

SI Table 9. Changes in mortality attributed to foods that are related to dietary risks and that are traded from exporting to importing regions, measured in changes in the number of deaths (deaths). The regions are grouped by income and include high-income countries (HIC), upper middle-income countries (UMC), lower middle-income countries (LMC), low-income countries (LIC), and an aggregate of all countries (World). An import-to-export ratio was calculated by dividing the mortality impacts of imports by those linked to exports, so that a value of greater than one indicates that food imports in a region were associated with greater health impacts than the region's exports in other regions.

Dietary risks	Exporter	Importer					Import/export ratio
		World	HIC	UMC	LMC	LIC	
all risks	World	-1,225,880	-715,823	-377,742	-122,684	-9,632	
	HIC	-369,159	-262,733	-64,467	-40,399	-1,560	1.94
	UMC	-614,340	-369,126	-193,149	-47,311	-4,754	0.61
	LMC	-210,436	-79,199	-100,906	-27,247	-3,084	0.58
	LIC	-31,946	-4,764	-19,220	-7,727	-235	0.30
fruits	World	-597,038	-363,058	-196,778	-34,379	-2,824	
	HIC	-150,303	-113,333	-29,809	-6,931	-230	2.42
	UMC	-349,323	-214,770	-114,138	-19,257	-1,158	0.56
	LMC	-93,836	-34,302	-51,396	-6,719	-1,420	0.37
	LIC	-3,576	-653	-1,435	-1,472	-16	0.79
vegetables	World	-380,217	-268,776	-83,137	-25,049	-3,256	
	HIC	-166,016	-143,318	-18,422	-3,826	-449	1.62
	UMC	-161,464	-95,628	-49,604	-14,174	-2,058	0.51
	LMC	-50,307	-28,829	-14,543	-6,228	-708	0.50
	LIC	-2,430	-1,001	-568	-820	-41	1.34
legumes	World	-98,163	-27,335	-33,107	-35,764	-1,957	
	HIC	-65,159	-17,162	-24,090	-23,167	-740	0.42
	UMC	-17,431	-6,255	-5,046	-5,108	-1,021	1.90
	LMC	-13,186	-3,571	-3,198	-6,303	-115	2.71
	LIC	-2,387	-347	-773	-1,186	-80	0.82
nuts	World	-299,852	-146,354	-118,544	-33,156	-1,797	
	HIC	-105,743	-71,426	-25,253	-8,844	-219	1.38
	UMC	-114,015	-59,297	-42,536	-11,562	-620	1.04
	LMC	-56,484	-12,848	-34,304	-8,470	-861	0.59
	LIC	-23,610	-2,783	-16,450	-4,280	-97	0.08
red meat	World	147,160	87,922	53,529	5,537	171	
	HIC	116,909	81,350	33,113	2,367	79	0.75
	UMC	27,005	6,272	17,944	2,713	76	1.98
	LMC	3,192	281	2,468	426	16	1.73
	LIC	54	19	4	31	0	3.17

Supplementary References

1 Dalin C, Wada Y, Kastner T, Puma MJ. Groundwater depletion embedded in international food trade. *Nature* 2017; **543**: 700–4.

2 Kastner T, Kastner M, Nonhebel S. Tracing distant environmental impacts of agricultural products from a consumer perspective. *Ecological Economics* 2011; **70**: 1032–40.

3 Miller RE, Blair PD. Input-output analysis: foundations and extensions. Cambridge University Press, 2009.

4 Carlson KM, Gerber JS, Mueller ND, *et al.* Greenhouse gas emissions intensity of global croplands. *Nature Climate Change* 2017; **7**: 63–8.

5 Murray CJL, Ezzati M, Lopez AD, Rodgers A, Vander Hoorn S. Comparative quantification of health risks: conceptual framework and methodological issues. *Population Health Metrics* 2003; **1**: 1.

6 Lim SS, Vos T, Flaxman AD, *et al.* A comparative risk assessment of burden of disease and injury attributable to 67 risk factors and risk factor clusters in 21 regions, 1990–2010: a systematic analysis for the Global Burden of Disease Study 2010. *The Lancet* 2012; **380**: 2224–60.

7 Forouzanfar MH, Alexander L, Anderson HR, *et al.* Global, regional, and national comparative risk assessment of 79 behavioural, environmental and occupational, and metabolic risks or clusters of risks in 188 countries, 1990–2013: a systematic analysis for the Global Burden of Disease Study 2013. *The Lancet* 2015; **386**: 2287–323.

8 Murray CJL, Ezzati M, Flaxman AD, *et al.* GBD 2010: design, definitions, and metrics. *Lancet* 2012; **380**: 2063–6.

9 Lozano R, Naghavi M, Foreman K, *et al.* Global and regional mortality from 235 causes of death for 20 age groups in 1990 and 2010: a systematic analysis for the Global Burden of Disease Study 2010. *The Lancet* 2012; **380**: 2095–128.

10 Afshin A, Micha R, Khatibzadeh S, Mozaffarian D. Consumption of nuts and legumes and risk of incident ischemic heart disease, stroke, and diabetes: a systematic review and meta-analysis. *The American Journal of Clinical Nutrition* 2014; : ajcn.076901.

11 Aune D, Keum N, Giovannucci E, *et al.* Nut consumption and risk of cardiovascular disease, total cancer, all-cause and cause-specific mortality: a systematic review and dose-response meta-analysis of prospective studies. *BMC medicine* 2016; **14**: 207.

12 Aune D, Giovannucci E, Boffetta P, *et al.* Fruit and vegetable intake and the risk of cardiovascular disease, total cancer and all-cause mortality–a systematic review and dose-response meta-analysis of prospective studies. *International Journal of Epidemiology* 2016; published online March 18.

13 Bechthold A, Boeing H, Schwedhelm C, *et al.* Food groups and risk of coronary heart disease, stroke and heart failure: A systematic review and dose-response meta-analysis of prospective studies. *Critical Reviews in Food Science and Nutrition* 2019; **59**: 1071–90.

14 Schwingshackl L, Hoffmann G, Lampousi AM, *et al.* Food groups and risk of type 2 diabetes mellitus: a systematic review and meta-analysis of prospective studies. *European Journal of Epidemiology* 2017; **32**: 363–75.

15 Schwingshackl L, Schwedhelm C, Hoffmann G, *et al.* Food groups and risk of colorectal cancer. *International Journal of Cancer* 2018; **142**: 1748–58.

16 Singh GM, Danaei G, Farzadfar F, *et al.* The Age-Specific Quantitative Effects of Metabolic Risk Factors on Cardiovascular Diseases and Diabetes: A Pooled Analysis. *PLOS ONE* 2013; **8**: e65174.

17 Micha R, Shulkin ML, Peñalvo JL, *et al.* Etiologic effects and optimal intakes of foods and nutrients for risk of cardiovascular diseases and diabetes: Systematic reviews and meta-analyses from the Nutrition and Chronic Diseases Expert Group (NutriCoDE). *PLOS ONE* 2017; **12**: e0175149.

18 GBD 2017 Diet Collaborators A, Sur PJ, Fay KA, *et al.* Health effects of dietary risks in 195 countries, 1990-2017: a systematic analysis for the Global Burden of Disease Study 2017. *Lancet (London, England)* 2019; **0**. DOI:10.1016/S0140-6736(19)30041-8.

19 Schwingshackl L, Knüppel S, Schwedhelm C, *et al.* Perspective: NutriGrade: A Scoring System to Assess and Judge the Meta-Evidence of Randomized Controlled Trials and Cohort Studies in Nutrition Research. *Advances in Nutrition: An International Review Journal* 2016; **7**: 994–1004.

20 World Cancer Research Fund/American Institute for Cancer Research. Diet, Nutrition, Physical Activity and Cancer: A Global Perspective. Continuous Update Project Expert Report. 2018.

21 Aune D, Norat T, Romundstad P, Vatten LJ. Dairy products and the risk of type 2 diabetes: a systematic review and dose-response meta-analysis of cohort studies. *The American Journal of Clinical Nutrition* 2013; **98**: 1066–83.

22 Aune D, Lau R, Chan DSM, *et al.* Dairy products and colorectal cancer risk: a systematic review and meta-analysis of cohort studies. *Annals of Oncology: Official Journal of the European Society for Medical Oncology* 2012; **23**: 37–45.

23 Mohan D, Mente A, Dehghan M, *et al.* Associations of Fish Consumption With Risk of Cardiovascular Disease and Mortality Among Individuals With or Without Vascular Disease From 58 Countries. *JAMA Internal Medicine* 2021; published online March 8. DOI:10.1001/jamainternmed.2021.0036.

Checklist of information that should be included in new reports of global health estimates

Item #	Checklist item	Reported on page #
**Objectives and funding**		
**1**	Define the indicator(s), populations (including age, sex, and geographic entities), and time period(s) for which estimates were made.	Attributable deaths in 2019 by country and five-year age group; see SI section 'Comparative risk assessment'
**2**	List the funding sources for the work.	Wellcome Trust; see abstract
**Data Inputs**		
For all data inputs from multiple sources that are synthesized as part of the study:		
**3**	Describe how the data were identified and how the data were accessed.	Trade data from FAOSTAT; mortality and population data from GBD; see main methods section
**4**	Specify the inclusion and exclusion criteria. Identify all ad-hoc exclusions.	No exclusions
**5**	Provide information on all included data sources and their main characteristics. For each data source used, report reference information or contact name/institution, population represented, data collection method, year(s) of data collection, sex and age range, diagnostic criteria or measurement method, and sample size, as relevant.	See main methods and SI sections 'Bilateral trade data' and 'Comparative risk assessment'
**6**	Identify and describe any categories of input data that have potentially important biases (e.g., based on characteristics listed in item 5).	See main methods and SI sections 'Bilateral trade data' and 'Comparative risk assessment'
For data inputs that contribute to the analysis but were not synthesized as part of the study:		
**7**	Describe and give sources for any other data inputs.	See main methods and SI sections 'Bilateral trade data' and 'Comparative risk assessment'
For all data inputs:		
**8**	Provide all data inputs in a file format from which data can be efficiently extracted (e.g., a spreadsheet rather than a PDF), including all relevant meta-data listed in item 5. For any data inputs that cannot be shared because of ethical or legal reasons, such as third-party ownership, provide a contact name or the name of the institution that retains the right to the data.	All inputs are made available as a supplementary data file (in spreadsheet format).
**Data analysis**		
**9**	Provide a conceptual overview of the data analysis method. A diagram may be helpful.	See main methods section
**10**	Provide a detailed description of all steps of the analysis, including mathematical formulae. This description should cover, as relevant, data cleaning, data pre-processing, data adjustments and weighting of data sources, and mathematical or statistical model(s).	See main methods and SI sections 'Bilateral trade data' and 'Comparative risk assessment'
**11**	Describe how candidate models were evaluated and how the final model(s) were selected.	There were no candidate models; the final model is described; see SI section 'Comparative risk assessment'
**12**	Provide the results of an evaluation of model performance, if done, as well as the results of any relevant sensitivity analysis.	The analysis follows standard formulae of comparative risk assessments; see SI section 'Comparative risk assessment'
**13**	Describe methods for calculating uncertainty of the estimates. State which sources of uncertainty were, and were not, accounted for in the uncertainty analysis.	See main methods and SI section 'Comparative risk assessment'
**14**	State how analytic or statistical source code used to generate estimates can be accessed.	See data sharing statement
**Results and Discussion**		
**15**	Provide published estimates in a file format from which data can be efficiently extracted.	All results are made available as a supplementary data file (in spreadsheet format).
**16**	Report a quantitative measure of the uncertainty of the estimates (e.g. uncertainty intervals).	See main methods and SI section 'Comparative risk assessment'
**17**	Interpret results in light of existing evidence. If updating a previous set of estimates, describe the reasons for changes in estimates.	See main discussion section.

**18**	Discuss limitations of the estimates. Include a discussion of any modelling assumptions or data limitations that affect interpretation of the estimates.	See main discussion section.
