# Supplementary material for: International food trade contributes to dietary risks and mortality at global, regional and national levels
Source: Nat Food. 2023 Oct 9;4(10):886–93. doi: 10.1038/s43016-023-00852-4 (PMC10589094; doi:10.1038/s43016-023-00852-4)
Supplement: Supplementary file 2 — Reporting Summary [file 43016_2023_852_MOESM2_ESM.pdf]

nature portfolio

Corresponding author(s): Marco Springmann

Last updated by author(s): Sep 5, 2023

Reporting Summary

Nature Portfolio wishes to improve the reproducibility of the work that we publish. This form provides structure for consistency and transparency in reporting. For further information on Nature Portfolio policies, see our Editorial Policies and the Editorial Policy Checklist.

Statistics

For all statistical analyses, confirm that the following items are present in the figure legend, table legend, main text, or Methods section.

n/a	Confirmed	
☒	☐	The exact sample size (*n*) for each experimental group/condition, given as a discrete number and unit of measurement
☒	☐	A statement on whether measurements were taken from distinct samples or whether the same sample was measured repeatedly
☒	☐	The statistical test(s) used AND whether they are one- or two-sided<br>*Only common tests should be described solely by name; describe more complex techniques in the Methods section.*
☒	☐	A description of all covariates tested
☒	☐	A description of any assumptions or corrections, such as tests of normality and adjustment for multiple comparisons
☐	☒	A full description of the statistical parameters including central tendency (e.g. means) or other basic estimates (e.g. regression coefficient) AND variation (e.g. standard deviation) or associated estimates of uncertainty (e.g. confidence intervals)
☒	☐	For null hypothesis testing, the test statistic (e.g. *F*, *t*, *r*) with confidence intervals, effect sizes, degrees of freedom and *P* value noted<br>*Give P values as exact values whenever suitable.*
☒	☐	For Bayesian analysis, information on the choice of priors and Markov chain Monte Carlo settings
☒	☐	For hierarchical and complex designs, identification of the appropriate level for tests and full reporting of outcomes
☒	☐	Estimates of effect sizes (e.g. Cohen's *d*, Pearson's *r*), indicating how they were calculated

Our web collection on statistics for biologists contains articles on many of the points above.

Software and code

Policy information about availability of computer code

Data collection	Data used for the analysis include trade data for the year 2019 from the Food and Agricultural Organization (accessible at https://www.fao.org/faostat/en/#data; accessed 3/2022), and mortality and population data from the Global Burden of Disease project for the year 2019 (accessible at: https://vizhub.healthdata.org/gbd-results/; accessed 3/2022). The data sources are open source.
Data analysis	We used an input-output algorithm for processing and analysing the trade data, and we used a comparative risk assessment framework for the health analysis. Both analyses are described in detail in Supplementary Information and the Supplementary GATHER checklist.

For manuscripts utilizing custom algorithms or software that are central to the research but not yet described in published literature, software must be made available to editors and reviewers. We strongly encourage code deposition in a community repository (e.g. GitHub). See the Nature Portfolio guidelines for submitting code & software for further information.

Data

Policy information about availability of data

All manuscripts must include a data availability statement. This statement should provide the following information, where applicable:

- Accession codes, unique identifiers, or web links for publicly available datasets
- A description of any restrictions on data availability
- For clinical datasets or third party data, please ensure that the statement adheres to our policy

All data produced in this study are available as a Supplementary Data File available on figshare via https://figshare.com/s/24b15c6b93caad07a758 and with the Digital Object Identifier (DOI) https://doi.org/10.25446/oxford.24085362. The codes for the trade and health analyses are described in detail in the Supplementary Information and the references cited therein. They are available upon request.

Field-specific reporting

Please select the one below that is the best fit for your research. If you are not sure, read the appropriate sections before making your selection.

☐ Life sciences ☒ Behavioural & social sciences ☐ Ecological, evolutionary & environmental sciences

For a reference copy of the document with all sections, see nature.com/documents/nr-reporting-summary-flat.pdf

Behavioural & social sciences study design

All studies must disclose on these points even when the disclosure is negative.

Study description	Quantitative modelling study based on bilateral trade flows and diet-related mortality associated with changes in dietary risks
Research sample	National level analysis with global coverage; the data sources used (on food trade, intake, mortality, and population) are nationally representative
Sampling strategy	The study design was a modelling study and therefore did not require sampling.
Data collection	We used publicly accessible data sources (FAOSTAT and GBD) for our analysis. Data used for the analysis include trade data for the year 2019 from the Food and Agricultural Organization (accessible at https://www.fao.org/faostat/en/#data; accessed 3/2022), and mortality and population data from the Global Burden of Disease project for the year 2019 (accessible at: https://vizhub.healthdata.org/gbd-results/; accessed 3/2022). The data sources are open source.
Timing	The data was annual and covered the year 2019. Data was collected in March 2022.
Data exclusions	Data of specific countries were excluded if one of three data sources (food intake, mortality, population) did not include data for that country.
Non-participation	No participants were involved in the study.
Randomization	The study design was a modelling study and therefore did not require randomization.

Reporting for specific materials, systems and methods

We require information from authors about some types of materials, experimental systems and methods used in many studies. Here, indicate whether each material, system or method listed is relevant to your study. If you are not sure if a list item applies to your research, read the appropriate section before selecting a response.

Materials & experimental systems

n/a	Involved in the study	
☒	☐	Antibodies
☒	☐	Eukaryotic cell lines
☒	☐	Palaeontology and archaeology
☒	☐	Animals and other organisms
☒	☐	Human research participants
☒	☐	Clinical data
☒	☐	Dual use research of concern

Methods

n/a	Involved in the study	
☒	☐	ChIP-seq
☒	☐	Flow cytometry
☒	☐	MRI-based neuroimaging
